# Supplementary material for: AI-based chest X-ray prioritization in the lung cancer diagnostic pathway: the LungIMPACT randomized controlled trial
Source: Nat Med. 2026 Mar 24;32(5):1737–44. doi: 10.1038/s41591-026-04253-5 (PMC13190311; doi:10.1038/s41591-026-04253-5)
Supplement: Supplementary file 1 — Reporting Summary [file 41591_2026_4253_MOESM1_ESM.pdf]

nature portfolio

Corresponding author(s): David R Baldwin

Last updated by author(s): 22/01/2026

Reporting Summary

Nature Portfolio wishes to improve the reproducibility of the work that we publish. This form provides structure for consistency and transparency in reporting. For further information on Nature Portfolio policies, see our Editorial Policies and the Editorial Policy Checklist.

Statistics

For all statistical analyses, confirm that the following items are present in the figure legend, table legend, main text, or Methods section.

n/a	Confirmed	
☐	☒	The exact sample size (*n*) for each experimental group/condition, given as a discrete number and unit of measurement
☐	☒	A statement on whether measurements were taken from distinct samples or whether the same sample was measured repeatedly
☐	☒	The statistical test(s) used AND whether they are one- or two-sided<br>*Only common tests should be described solely by name; describe more complex techniques in the Methods section.*
☐	☒	A description of all covariates tested
☐	☒	A description of any assumptions or corrections, such as tests of normality and adjustment for multiple comparisons
☐	☒	A full description of the statistical parameters including central tendency (e.g. means) or other basic estimates (e.g. regression coefficient) AND variation (e.g. standard deviation) or associated estimates of uncertainty (e.g. confidence intervals)
☐	☒	For null hypothesis testing, the test statistic (e.g. *F*, *t*, *r*) with confidence intervals, effect sizes, degrees of freedom and *P* value noted<br>*Give P values as exact values whenever suitable.*
☒	☐	For Bayesian analysis, information on the choice of priors and Markov chain Monte Carlo settings
☒	☐	For hierarchical and complex designs, identification of the appropriate level for tests and full reporting of outcomes
☐	☒	Estimates of effect sizes (e.g. Cohen's *d*, Pearson's *r*), indicating how they were calculated

Our web collection on statistics for biologists contains articles on many of the points above.

Software and code

Policy information about availability of computer code

Data collection	qXR (v 4.0, Qure.ai Technologies Limited, UK), is a class IIb CE certified deep learning algorithm and was used as the CXR AI intervention qXR is a proprietary, MDR CE-class IIb commercial product by Qure.ai. Source code and model weights are not open-source and are not publicly downloadable. Data was collected electronically and manually verified for CT data and lung cancer data.
Data analysis	All data were analysed with Stata v19.5 (StataCorp, College Station, TX, USA), according to the pre-specified analysis plan. Code has been deposited on GitHib

For manuscripts utilizing custom algorithms or software that are central to the research but not yet described in published literature, software must be made available to editors and reviewers. We strongly encourage code deposition in a community repository (e.g. GitHub). See the Nature Portfolio guidelines for submitting code & software for further information.

Data

Policy information about availability of data

All manuscripts must include a data availability statement. This statement should provide the following information, where applicable:

- Accession codes, unique identifiers, or web links for publicly available datasets
- A description of any restrictions on data availability
- For clinical datasets or third party data, please ensure that the statement adheres to our policy

Data were collected and pseudonymised for analysis. The non-consenting nature of the study means that access to the data held by the sponsor requires an additional ethical approval. Access to deannonymised data is only available to the clinical teams at each partcipating site.

Research involving human participants, their data, or biological material

Policy information about studies with human participants or human data. See also policy information about sex, gender (identity/presentation), and sexual orientation and race, ethnicity and racism.

Reporting on sex and gender	Sex data only collected; Female 24,709 (53.7%) in intervention and 25,352 (53.6%) in control. A subgroup analysis was performed and is included in a supplementary upload
Reporting on race, ethnicity, or other socially relevant groupings	No data were collected for race, ethnicity or social grouping.
Population characteristics	Mean age 59.1 (SD 17.4), same for intervention and control; 53.6% female, same for intervention and control
Recruitment	Participants aged 18 years or over, who were attending for a primary care requested CXR, were block randomised by day and site, into immediate AI prioritisation of reporting versus no AI prioritisation. Pre-allocation to intervention or routine care was via a random sampling on a 1:1 randomisation method for Monday to Friday routine imaging whole day sessions.
Ethics oversight	East of England Cambridge East REC; 23/EE/0014 21st February 2023

Note that full information on the approval of the study protocol must also be provided in the manuscript.

Field-specific reporting

Please select the one below that is the best fit for your research. If you are not sure, read the appropriate sections before making your selection.

☒ Life sciences ☐ Behavioural & social sciences ☐ Ecological, evolutionary & environmental sciences

For a reference copy of the document with all sections, see nature.com/documents/nr-reporting-summary-flat.pdf

Life sciences study design

All studies must disclose on these points even when the disclosure is negative.

Sample size	Using data from previous work,(20) the median time to lung cancer diagnosis was 63 days in the standard reporting group and using a conservative reduction of 10 days, we calculated that 265 cases per group would be needed to detect a difference with 95% power. The expected prevalence of lung cancer in this cohort (primary care referrals for CXR) is 0.6%(20) and thus 100,000 CXRs were judged sufficient (600 cancer cases). For time to CT, the NHS Diagnostic Imaging Dataset records a median 15 days between request and CT, but data are not segregated between urgent scans and those that are routine or for follow up(21). The mean and standard deviation of time between abnormal CXR and CT chest are not known. Cohen's d (effect size) was used to estimate sample size with power 0.1, 0.3 and 0.5 (considered small, moderate and large effect size)(22). Assuming approximately equal distribution of the number of scans taken, 4,000 CT scans were found to provide adequate power to detect even the smallest effect size (Cohen's d = 0.1). A clinically meaningful difference in time from abnormal CXR to CT chest as 3 days was defined, using the maximum time recommended in the NOLCP.
Data exclusions	4,405 CXRs excluded; data compliance issue/technical failure n=1,382, weekend CXR n=3,023
Replication	A major strength of the trial is the randomised controlled design without the need to take individual consent from participants. This meant that there was minimal influence on the normal clinical pathway and more equitable inclusion of groups underrepresented in research. Information in the radiology departments made it clear that there was an option to opt out of the study but there was only one. In addition, the trial was multicentre and included different sizes, geographies and catchment demographics of NHS hospitals.
Randomization	Participants aged 18 years or over, who were attending for a primary care requested CXR, were block randomised by day and site, into immediate AI prioritisation of reporting versus no AI prioritisation. Pre-allocation to intervention or routine care was via a random sampling on a 1:1 randomisation method for Monday to Friday routine imaging whole day sessions.
Blinding	Blinding was not possible, the intervention was radiology worklist prioritisation. However radiology clinicians were blinded to the randomsiation schedule/sequence.

April 2023

Reporting for specific materials, systems and methods

We require information from authors about some types of materials, experimental systems and methods used in many studies. Here, indicate whether each material, system or method listed is relevant to your study. If you are not sure if a list item applies to your research, read the appropriate section before selecting a response.

Materials & experimental systems

n/a	Involved in the study
☒	☐ Antibodies
☒	☐ Eukaryotic cell lines
☒	☐ Palaeontology and archaeology
☒	☐ Animals and other organisms
☐	☒ Clinical data
☒	☐ Dual use research of concern
☒	☐ Plants

Methods

n/a	Involved in the study
☒	☐ ChIP-seq
☒	☐ Flow cytometry
☒	☐ MRI-based neuroimaging

Clinical data

Policy information about clinical studies

All manuscripts should comply with the ICMJE guidelines for publication of clinical research and a completed CONSORT checklist must be included with all submissions.

Clinical trial registration

Trial registration ISRCTN 78987039 7th March 2023

Study protocol

Available at trial registration https://www.isrctn.com/ISRCTN78987039

Data collection

Between 17th July 2023 and 31st December 2024, 97,731 CXRs were performed across five diverse NHS Trusts, with a geographical spread and a mix of general/tertiary and high/low volume centres. Patients were followed up until 5th June 2025. The mean age of the study population was 59 years and 46% were male. University Hospitals of Leicester (UHL) performed 54% of the study CXRs, Nottingham University Hospitals (NUH) 15%, East Sussex and North Essex Foundation Trust (ESNEFT) 11%, University College London Hospital (UCLH) 11% and University Hospitals of Birmingham (UHB) 9% (Table S1)

Outcomes

The primary outcomes were:
1. The difference in time (in days from CXR request) to the diagnosis of lung cancer for patients who have CXRs with AI support at the time of CXR acquisition and prioritisation for immediate review and those that have no immediate read but the AI read is available at the time of reporting.
2. The difference in time (in days from CXR request) to CT for patients who have CXRs with AI support at the time of CXR acquisition and prioritisation for immediate review and those that have no immediate read but the AI read is available at the time of reporting.
The secondary outcomes were:
1. Days to urgent lung cancer referral as defined by time between CXR acquisition and lung cancer referral (2WW)
2. Days to treatment start for lung cancer patients
3. Agreement between qXR and human readers for present/absent findings on CXRs referred from primary care, by each classification
4. Number of urgent lung cancer (2WW) referrals
5. Incidence of lung cancer
6. Stage of lung cancer at diagnosis
7. Algorithm sensitivity, specificity and agreement between report and AI (Kappa)

Plants

Seed stocks

Report on the source of all seed stocks or other plant material used. If applicable, state the seed stock centre and catalogue number. If plant specimens were collected from the field, describe the collection location, date and sampling procedures.

Novel plant genotypes

Describe the methods by which all novel plant genotypes were produced. This includes those generated by transgenic approaches, gene editing, chemical/radiation-based mutagenesis and hybridization. For transgenic lines, describe the transformation method, the number of independent lines analyzed and the generation upon which experiments were performed. For gene-edited lines, describe the editor used, the endogenous sequence targeted for editing, the targeting guide RNA sequence (if applicable) and how the editor was applied.

Authentication

Describe any authentication procedures for each seed stock used or novel genotype generated. Describe any experiments used to assess the effect of a mutation and, where applicable, how potential secondary effects (e.g. second site T-DNA insertions, mosiacism, off-target gene editing) were examined.
